# Myocardial changes on 3T cardiovascular magnetic resonance imaging in response to haemodialysis with fluid removal

**DOI:** 10.1186/s12968-021-00822-4

**Published:** 2021-11-11

**Authors:** Alastair J. Rankin, Kenneth Mangion, Jennifer S. Lees, Elaine Rutherford, Keith A. Gillis, Elbert Edy, Laura Dymock, Thomas A. Treibel, Aleksandra Radjenovic, Rajan K. Patel, Colin Berry, Giles Roditi, Patrick B. Mark

**Affiliations:** 1 Institute of Cardiovascular and Medical Sciences, University of Glasgow, 126 University Place, Glasgow, G12 8TA UK; 2 Renal and Transplant Unit, NHS Greater Glasgow and Clyde, Glasgow, UK; 3 Clinical Research Imaging, NHS Greater Glasgow and Clyde, Glasgow, UK; 4 Institute for Cardiovascular Sciences and Barts Heart Centre, University College London, London, UK; 5 Department of Radiology, NHS Greater Glasgow and Clyde, Glasgow, UK

**Keywords:** End-stage kidney disease, Haemodialysis, Cardiovascular, Magnetic resonance imaging, Left ventricular hypertrophy

## Abstract

**Background:**

Mapping of left ventricular (LV) native T1 is a promising non-invasive, non-contrast imaging biomarker. Native myocardial T1 times are prolonged in patients requiring dialysis, but there are concerns that the dialysis process and fluctuating fluid status may confound results in this population. We aimed to assess the changes in cardiac parameters on 3T cardiovascular magnetic resonance (CMR) before and after haemodialysis, with a specific focus on native T1 mapping.

**Methods:**

This is a single centre, prospective observational study in which maintenance haemodialysis patients underwent CMR before and after dialysis (both scans within 24 h). Weight measurement, bio-impedance body composition monitoring, haemodialysis details and fluid intake were recorded. CMR protocol included cine imaging and mapping native T1 and T2.

**Results:**

Twenty-six participants (16 male, 65 ± 9 years) were included in the analysis. The median net ultrafiltration volume on dialysis was 2.3 L (IQR 1.8, 2.5), resulting in a median weight reduction at post-dialysis scan of 1.35 kg (IQR 1.0, 1.9), with a median reduction in over-hydration (as measured by bioimpedance) of 0.75 L (IQR 0.5, 1.4). Significant reductions were observed in LV end-diastolic volume (− 25 ml, p = 0.002), LV stroke volume (− 13 ml, p = 0.007), global T1 (21 ms, p = 0.02), global T2 (− 1.2 ms, p = 0.02) following dialysis. There was no change in LV mass (p = 0.35), LV ejection fraction (p = 0.13) or global longitudinal strain (p = 0.22). On linear regression there was no association between baseline over-hydration (as defined by bioimpedance) and global native T1 or global T2, nor was there an association between the change in over-hydration and the change in these parameters.

**Conclusions:**

Acute changes in cardiac volumes and myocardial native T1 are detectable on 3T CMR following haemodialysis with fluid removal. The reduction in global T1 suggests that the abnormal native T1 observed in patients on haemodialysis is not entirely due to myocardial fibrosis.

**Supplementary Information:**

The online version contains supplementary material available at 10.1186/s12968-021-00822-4.

## Introduction

Patients with chronic kidney disease (CKD) are at a greatly increased risk of cardiovascular disease (CVD) [1]. This risk increases with severity of CKD [2], such that patients with CKD stage 5 are 3–4 times more likely to experience a cardiovascular event than age-standardized patients without CKD [3]. In patients with kidney disease receiving dialysis, CVD remains the single most common cause of death, accounting for between 25 and 40% of all deaths [4–6]. CKD results in a unique cardiovascular phenotype; with relatively fewer deaths due to atherosclerotic processes and more due to sudden cardiac death and heart failure [4–8]. Cardiomyopathy of CKD, often called ‘uraemic cardiomyopathy’, refers to a specific pattern of myocardial fibrosis, left ventricular (LV) hypertrophy and diastolic dysfunction, which is found in patients with CKD and forms the pathological basis for this unique CVD phenotype [9–11].

Cardiovascular magnetic resonance imaging (CMR) is established as the reference method for imaging uraemic cardiomyopathy [12]. Previous studies using gadolinium-enhanced CMR demonstrated the presence of myocardial fibrosis in patients on dialysis [13], and its association with poor survival [14]. However, the discovery of the association between gadolinium based contrast media in patients with CKD and the development of the very rare disease nephrogenic systemic fibrosis curtailed further research using this technique [15, 16]. There is a pressing need for an alternative marker of uraemic cardiomyopathy, further intensified by the observation that regression of LV mass in isolation may not be robustly associated with improved CVD outcomes in patients with CKD [17]. Attempts to identify reliable imaging biomarkers in this population are hampered by the potential confounding influence of the dialysis process itself and fluctuating fluid status.

Native T1 mapping is a non-contrast technique that estimates myocardial longitudinal relaxation times (ms) and reflects changes in extra- and intra-cellular compartments. Myocardial T1 is commonly affected by changes in collagen (fibrosis), water (oedema), iron deposition (haemochromatosis, myocardial haemorrhage) and lipids (Anderson-Fabry’s disease) [18]. In addition, native T1 mapping has been shown to differentiate dialysis patients from both healthy [19] and co-morbid controls [20], with excellent inter-observer reproducibility [20, 21]. Outside of the CKD population, T1 mapping has been shown to correlate well with myocardial fibrosis in other disease states [12, 22, 23]. However, the major concern with using native T1 mapping in dialysis patients is the potential confounding influence on the T1 signal of changing tissue oedema resulting from the large intra-dialytic fluid fluctuations that are typical of patients on intermittent haemodialysis [24]. A previous study using 1.5T CMR observed small, but detectable, differences in native T1 times immediately after haemodialysis [25]. In the present study we assess the myocardial changes on 3T CMR in response to haemodialysis with fluid removal, with a particular interest in native T1 to inform its potential suitability as a surrogate outcome measure in future therapeutic trials. We also explored the potential bias of dialysis timing in relation to the clinical applicability of 3T CMR.

## Materials and methods

### Participants

Participants were aged > 40 years and were established on regular, day-time hospital-based haemodialysis for at least 6 months. Participants were eligible for inclusion if they had a history of recurrent fluid overload (defined as requirement for ultrafiltration volumes of at least 1.5 L mean fluid removal over the preceding 3 dialysis sessions) and without heart failure (defined as no previous clinical diagnosis of heart failure or with preserved LV ejection fraction (LVEF) (> 50%) on their most recent transthoracic echocardiogram). Participants had to be able to comply with study procedures, self-report an ability to lie flat for 1 h and provide informed consent. Exclusion criteria included standard contra-indications to CMR and contraindications to iodine based radiological contrast (to facilitate a sub-study comparing CMR with a novel contrast CT technique) [26, 27]. The study was prospectively registered at clinicaltrials.gov (NCT03704701). Favourable ethical opinion was granted by the West of Scotland Research Ethics Committee 1 (Ref: 18/WS/0138, 13th August 2018). All study procedures were carried out in accordance with local guidelines and regulations and with respect to the Declaration of Helsinki.

### Study protocol

**Fig. 1:**
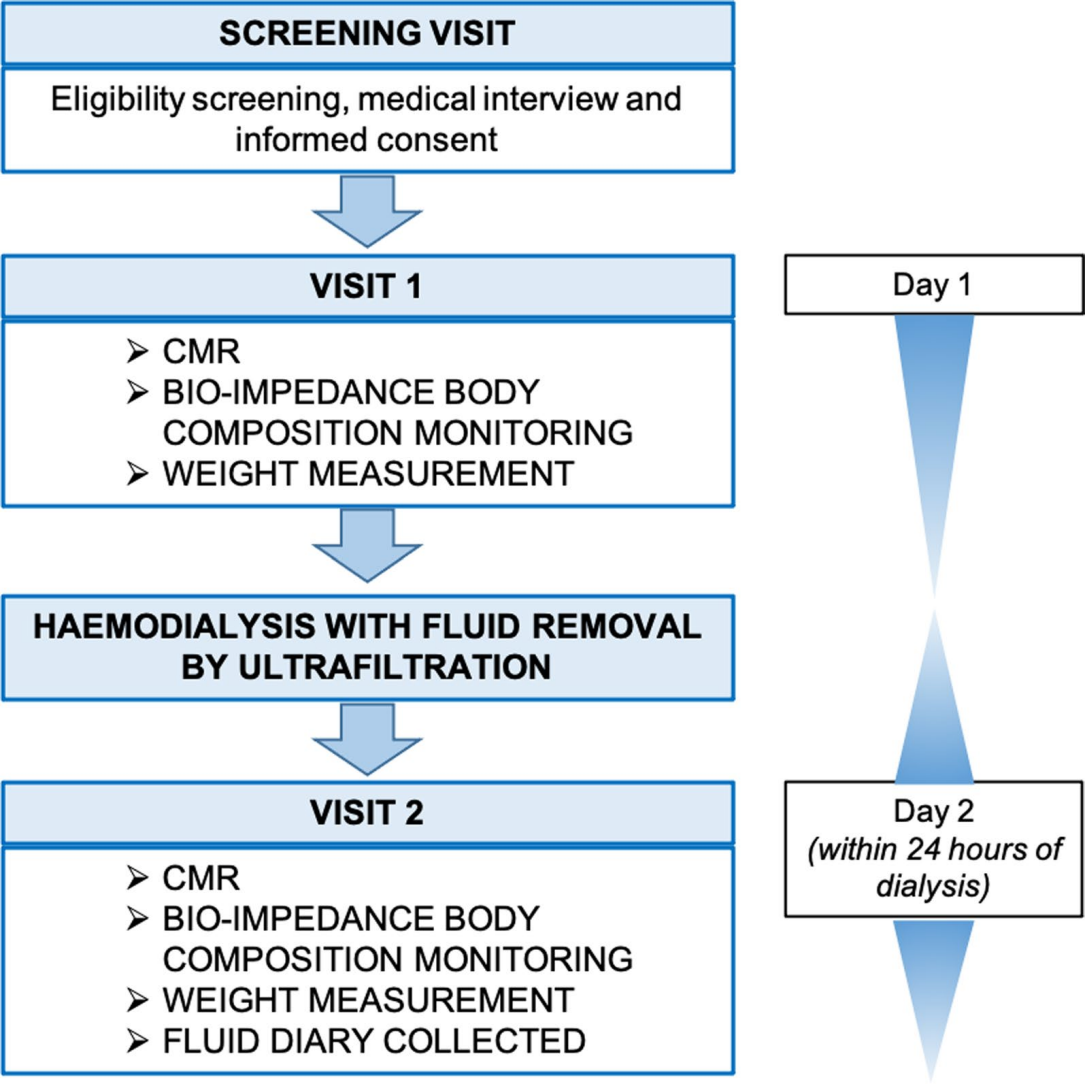
Flow chart depicting study procedures

This single centre observational study consisted of 2 visits (Fig. 1). Visit 1 occurred before a participant’s routine dialysis session. Where possible, this occurred at the end of a participant’s ‘long’, or two-day, gap, i.e., on a Monday for participants on a Monday, Wednesday, Friday dialysis schedule. Participants on a morning dialysis schedule attended visit 1 the afternoon before dialysis. Between visits 1 and 2 participants were asked to consume food and drink as they normally would but to document what they had taken. Participants would then attend their routine haemodialysis session which was performed as per usual clinical practice. Details of the dialysis session were recorded including duration, ultrafiltration volume, settings and medications administered. Visit 2 occurred after dialysis. Participants on an afternoon dialysis schedule attended visit 2 the following morning. At each visit, weight measurement, bioimpedance body composition monitoring (using a Fresenius Body Composition Monitor, Fresenius Medical Care, Hong Kong as per manufacturer’s instructions), blood tests and CMR were performed.

### CMR image acquisition

CMR acquisition was performed at the Clinical Research Imaging Facility of the Queen Elizabeth University Hospital in Glasgow using a 3 T CMR scanner (PRISMA, Siemens Healthineers, Erlangen, Germany) with an 18-channel surface coil placed anteriorly and a 32-channel spine coil placed posteriorly. Following the acquisition of localiser images, balanced steady state free precession sequences were used to acquire LV cine imaging in three long axis planes, followed by a short axis stack from the apex to the atrio-ventricular ring, each with 25 phases. Images were obtained using retrospective electrocardiogram (ECG)-gating at end-expiration. Where participants were unable to breath-hold or had cardiac arrhythmia, compressed sensing (CS cardiac Cine, Siemens Healthineers) was used to allow real-time acquisition. Typical scan parameters were: field of view (FOV) 340 × 286 mm, slice thickness 7 mm with 3 mm gap in short axis stack, repetition time (TR)—41.4 ms, echo time (TE) 1.51 ms, flip angle 50°, voxel size 1.33 × 1.33 × 7 mm.

For native T1 and T2 mapping, basal, mid and apical short axis views were acquired using SiemensMyoMaps sequences. For native T1, non-contrast, motion-corrected, optimized, modified Look-Locker inversion recovery sequences were used with the following typical parameters: FOV 340 × 272 mm, slice thickness 6.0 mm, voxel size: 1.9 × 1.9 × 6.0 mm, TR 272 ms, TE 1.12 ms, flip angle 35 degrees, minimum T1 100 ms, inversion-time increment 80 ms, bandwidth 1085 Hertz/pixel. For T2 mapping, three T2 weighted measurements were acquired followed by an automated exponential fit for each pixel after respiratory motion correction. The imaging used a T2-prepared single shot balanced steady state free precession readout with T2 preparation times (TE) = 0, 25, and 55 ms with a recovery period of 3 heartbeats between measurements. Typical protocol parameters for T2 mapping were: FOV 360 × 270 mm, slice thickness 8 mm, matrix 192 × 108, spatial resolution 1.9 × 2.5 mm, TR 207.39 ms, TE 1.32 ms, flip angle 12 degrees, bandwidth 1184 Hz/pixel.

### CMR image analysis

**Fig. 2:**
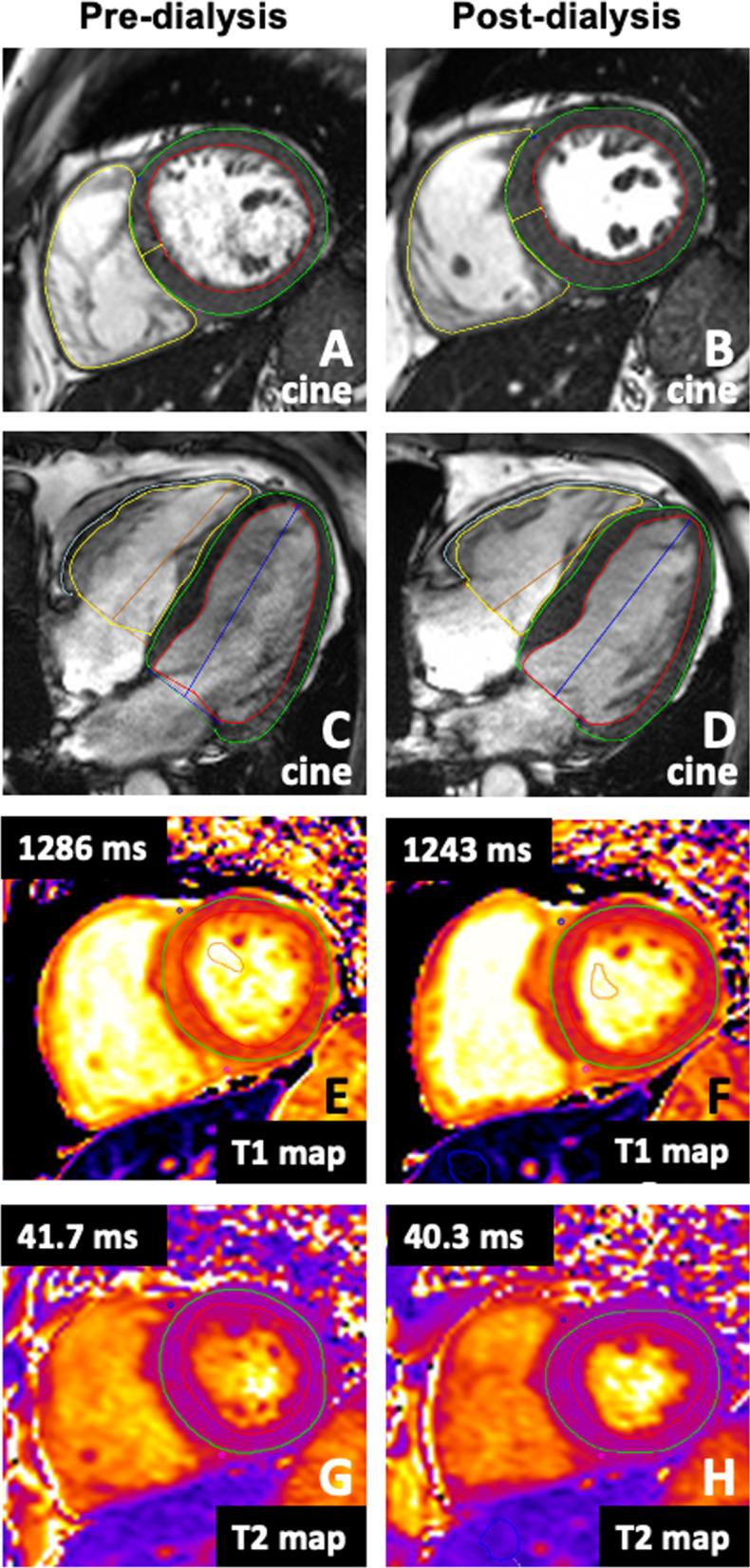
Representative 3T CMR images of mid- left ventricle end-diastolic short axis stack cine (**A**, **B**), end-diastolic horizontal long axis cine (**C**, **D**), native T1 mapping (**E**, **F**) and native T2 mapping (**G**, **H**) acquired before and after dialysis. In this representative participant, global T1 and global T2 reduced following 4 h of haemodialysis with 2.3 L ultrafiltration

All CMR scans were subject to a clinical report for clinical governance purposes. Research CMR analysis was performed utilizing dedicated CMR software (cvi42, version 5.10, Circle Cardiovascular Imaging, Calgary, Alberta, Canada)). Routinely reported CMR measures of LV and right ventricular (RV) function were carried out according to current guidelines [28]. Parameters of myocardial mass and volumes were not indexed to body surface area to avoid confounding impact of weight changes falsely adjusting body surface area and is acceptable given the analysis of within-subject comparisons. Ventricular endocardial and epicardial contours were manually drawn at end-diastole (Fig. 2). LV endocardial contours were drawn at end-systole, which was deemed to be the phase with the smallest blood pool cavity. Papillary muscles were excluded from myocardial mass and included in volumes. LV thickness was recorded as the maximum septal thickness measured perpendicular to the cavity on a short-axis mid-chamber view, at the approximate level of the mitral valve leaflet tips. Global LV strain (circumferential, longitudinal, and radial) and global RV strain (longitudinal and radial) were derived using the software’s tissue tracking module to determine peak values for each parameter. Atrial volumes were manually drawn on 4-chamber horizontal long axis views at atrial systole and diastole (defined with respect to mitral valve closure) to report maximum and minimum right atrial (RA) volumes and atrial emptying fraction. For left atrial (LA) measurements, the vertical long axis views were additionally contoured to report biplanar derived values. For T1 and T2 measurements, scanner derived maps were used. Epi- and endocardial borders were manually drawn on each basal, mid and apical map. Areas of obvious artefact were excluded from regions of interest (ROI) and care was taken to include only myocardial tissue with a 10% epi- and endocardial offset applied. Global values were derived by averaging results from all three short axis slices. Septal values were reported as the mean of segments 2, 3, 8, 9 and 14 as per the American Heart Association’s 16 segment model [29]. For blood pool T1 and T2, ROIs were drawn within the LV cavity on the mid-LV map, with care taken to avoid artefact and papillary muscles. Additional ROIs were manually drawn on a representative area of skeletal muscle, with the pectoralis major muscle used preferentially. A further ROI was drawn within a homogenous region within the right lobe of liver. The primary observer (AJR) batch analysed all CMRs in a random order and was blinded to participant identity and whether the scan was pre-or post-dialysis. A second independent observer (KM) analysed a random sample of > 20% of the cohort to assess inter-observer variability. As a post-hoc experiment, a T1MES phantom [30] was scanned on consecutive days at times that replicated the study schedule to assess inter-study T1 variability.

### Statistical analysis

Continuous data with a normal distribution are presented as mean ± standard deviation (SD), and median and interquartile range (IQR) for skewed data, with normality defined according to Shapiro–Wilk test. Pre and post dialysis CMR values were compared using paired t-tests and Wilcoxon singed rank tests accordingly. Linear regression and multiple regression were used to compare change in CMR parameters according to baseline variables. Repeated measures MANCOVA was used to account for covariates in the comparison of myocardial native T1 before and after dialysis. Intra- and interobserver variability was assessed by the intra-class correlation (ICC) coefficient (two-way mixed effect, average measures). Statistical analysis was performed, and figures created, using SPSS (version 27, Statistical Package for the Social Sciences, International Business Machines, Inc., Armonk, New York, USA).

### Sample size

A prospective sample size calculation determined that a total of 9 participants would be required to detect a 2.5% difference in native T1 times with 80% power and alpha 0.05 based on previously published data [19, 21]. A total of 22 participants would be sufficient to detect a 1.5% difference. A target of 30 participants was set to allow drop out and to facilitate a pre-specified sub-study (clinicaltrials.gov NCT03704701).

## Results

### Participant characteristics

Twenty-eight participants were recruited between 19th October 2018 and 9th March 2020. Recruitment was stopped early (target n = 30) due to the COVID-19 pandemic. Two participants withdrew consent prior to any study procedures leaving 26 for analysis, of whom 16 (61.5%) were male, 22 (84.6%) were white and age 64.7 ± 9.4 years. Median duration of kidney replacement therapy at time of recruitment was 2.0 (1.3, 4.0) years. Multi-morbidity was prevalent in the cohort with a mean Charlson Comorbidity index of 6 (mean modified Charlson Comorbidity Index of 3) [31, 32]. The median duration of haemodialysis treatment session was 4.0 h (4.0, 5.0) with a mean blood flow of 265 (± 32) ml/min. Eighteen (69.2%) participants followed an afternoon dialysis schedule and underwent dialysis median 2.5 (2.0, 2.8) hours after their first CMR, with repeat CMR at median 15.3 (14.8, 16.7) hours after completion of dialysis. The remaining 8 (30.8%) participants followed a morning dialysis schedule and underwent dialysis at median 16 (14.7, 16.2) hours after their first CMR, with a repeat CMR 1.5 (1.2, 2.7) hours after completing dialysis. For 23 (88.5%) participants, visit 1 took place after their ‘long gap’ between dialysis sessions (i.e., pre-dialysis on a Monday for a patient on a Monday, Wednesday, Friday dialysis schedule). Additional baseline characteristics are detailed in Additional file 1: Table S1. In 6 participants, clinically significant incidental findings were detected, including 2 cancers requiring treatment (Additional file 1: Table S2).

### Fluid status

All participants had a history of recurrent fluid overload with mean ultrafiltration volume of 2.2 L (± 0.4) from the preceding 3 dialysis sessions prior to recruitment. At visit 1 (pre-dialysis), 12 participants had demonstrable pitting oedema. 1 participant was unable to undergo bioimpedance monitoring for multifactorial reasons (body habitus, immobility, skin emollient). Of the remaining 25 participants, the median over-hydration was + 0.4 L (− 2.8, + 3.5), with 10 participants measuring as volume deplete pre-dialysis. The median net ultrafiltration volume on dialysis was 2.3 L (1.8, 2.5) at a mean rate of 6 mL/kg/h (± 1.7). Five participants experienced symptomatic intradialytic hypotension requiring adjustment of their dialysis prescription. Between visit 1 (pre-dialysis) and visit 2 (post-dialysis), the median estimated fluid intake was 0.9 L (0.6, 1.0). At visit 2 (post-dialysis), the median reduction in body weight was 1.4 kg (1.0, 1.9), with a median reduction in over-hydration of 0.8 L (0.5, 1.4). No participants gained weight between visit 1 and visit 2, albeit 2 participants’ weight did not change. According to bioimpedance monitoring, 3 participants increased their over-hydration between visits (range 0.2–0.4 L).

### CMR parameters pre- and post-dialysis

**Fig. 3:**
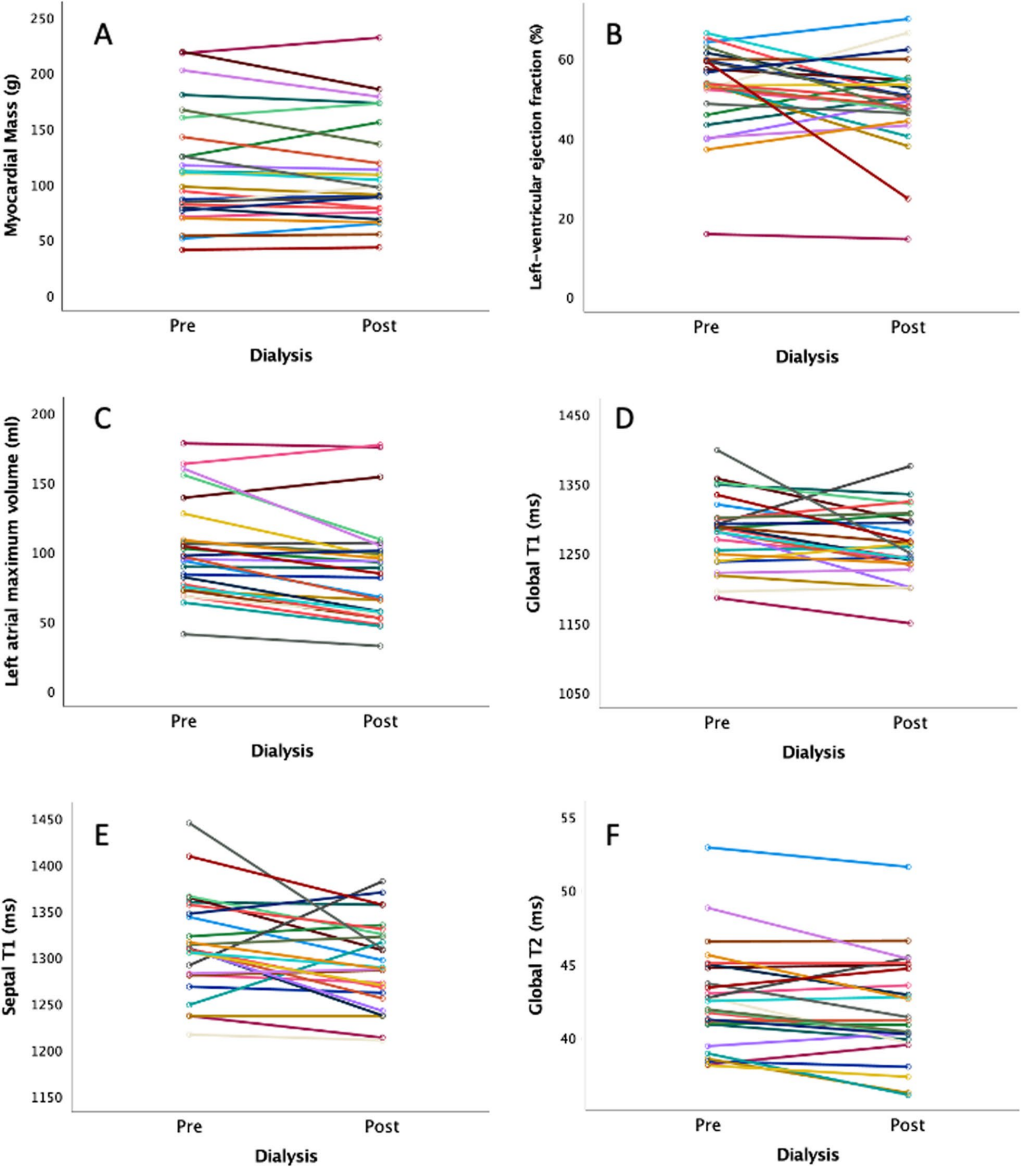
Within subject changes pre- and post- dialysis for left ventricular mass (**A**), left ventricular ejection fraction (**B**), left atrial maximum volume (**C**), global T1 times (**D**), septal T1 times (**E**), and global T2 times (**F**)

Table 1 shows the CMR results before and after dialysis. Notable findings include a significant reduction in LV end-diastolic volume, LV stroke volume, RV stroke volume, LA volumes, global circumferential strain, global native T1, septal native T1 and global T2 following dialysis. There was no change in LV mass, LV or RV ejection fraction or global longitudinal strain (Table 1). Figure 3 shows within-subject changes for LV mass, LVEF, LA maximum volume, global T1, septal T1 and global T2. The intra- and interobserver reproducibility for global T1 was excellent with ICC of 0.989 and 0.949, respectively. Additional intra- and interobserver reproducibility results are included in Additional file 1: Table S3.

**Table 1:** Cardiovascular Magnetic resonance (CMR) parameters pre- and post-dialysis

CMR parameter	Pre-dialysis	Post-dialysis	p-value
LV myocardial mass (g)	103.8 (78.8, 142.4)	97.5 (78.2, 136.0)	0.35
LV end diastolic volume (ml)	185 (159, 229)	160 (152, 220)	0.002
LV end systolic volume (ml)	88 (71, 113)	84 (69, 111)	0.81
LV stroke volume (ml)	103 (± 29)	90 (± 30)	0.007
LV ejection fraction (%)	53.6 (48.6, 59.5)	49.8 (46.2, 54.5)	0.13
LV global longitudinal strain (%)	− 13.8 (± 3.3)	− 13.1 (± 3.6)	0.22
LV global circumferential strain (%)	− 16.3 (− 19.5, − 14.0)	− 15.1 (− 16.9, − 13.4)	0.03
LV global radial strain (%)	22.2 (± 6.7)	20.7 (± 7.1)	0.18
LV thickness (mm)	10.2 (8.4, 12.2)	10.6 (8.8, 12.3)	0.44
RV end diastolic volume (ml)	161 (133, 184)	136 (128, 171)	< 0.001
RV end systolic volume (ml)	67 (56, 82)	62 (53, 75)	0.66
RV stroke volume (ml)	98 (± 30)	84 (± 26)	< 0.001
RV ejection fraction (%)	56.9 (± 10.5)	53.8 (± 12.6)	0.05
RV global longitudinal strain (%)	− 22.5 (± 5.9)	− 22.4 (± 6.8)	0.88
RV global radial strain (%)	48.3 (37.2, 66.7)	49.8 (40.0, 71.7)	0.77
Minimum LA volume (ml)	44 (28, 70)	40 (22, 70)	0.001
Maximum LA volume (ml)	96 (75, 108)	86 (57, 101)	< 0.001
Minimum RA volume (ml)	30 (20, 44)	29 (22, 41)	0.95
Maximum RA volume (ml)	60 (49, 77)	54 (45, 75)	0.09
Global native T1 (ms)	1283 (± 51)	1262 (± 49)	0.02
Septal native T1 (ms)	1313 (± 54)	1293 (± 47)	0.04
Blood pool native T1 (ms)	1957 (± 68)	1935 (± 73)	0.08
Skeletal muscle native T1 (ms)	1218 (± 65)	1210 (± 73)	0.60
Liver native T1 (ms)	686 (± 157)	679 (± 146)	0.45
Global T2 (ms)	42.2 (40.9, 44.8)	41.0 (39.9, 44.7)	0.02
Blood pool T2 (ms)	101.1 (± 20.9)	111.0 (± 24.5)	0.06
Skeletal muscle T2 (ms)	32.2 (± 2.1)	30.8 (± 3.0)	0.03
Liver T2 (ms)	21.6 (19.9, 23.5)	21.3 (20.1, 22.4)	0.81

Displayed as mean, standard deviation and paired t-test for variables with a normal distribution, and median, interquartile range and Wilcoxon signed rank test for those with a skewed distribution

*The scanner-specific reference range for myocardial native global T1 in healthy subjects is mean (range)* 1170 ms (1107–1234) and global T2 is mean 39.5 ms (34.7–44.3) (*unpublished data*, correspondence from Dr Kenneth Mangion and Dr Andrew Morrow)

*LV* left ventricular, *RV* right ventricular, *LA* left atrial, *RA* right atrial

### Change in native T1 and T2 by fluid status

**Fig. 4:**
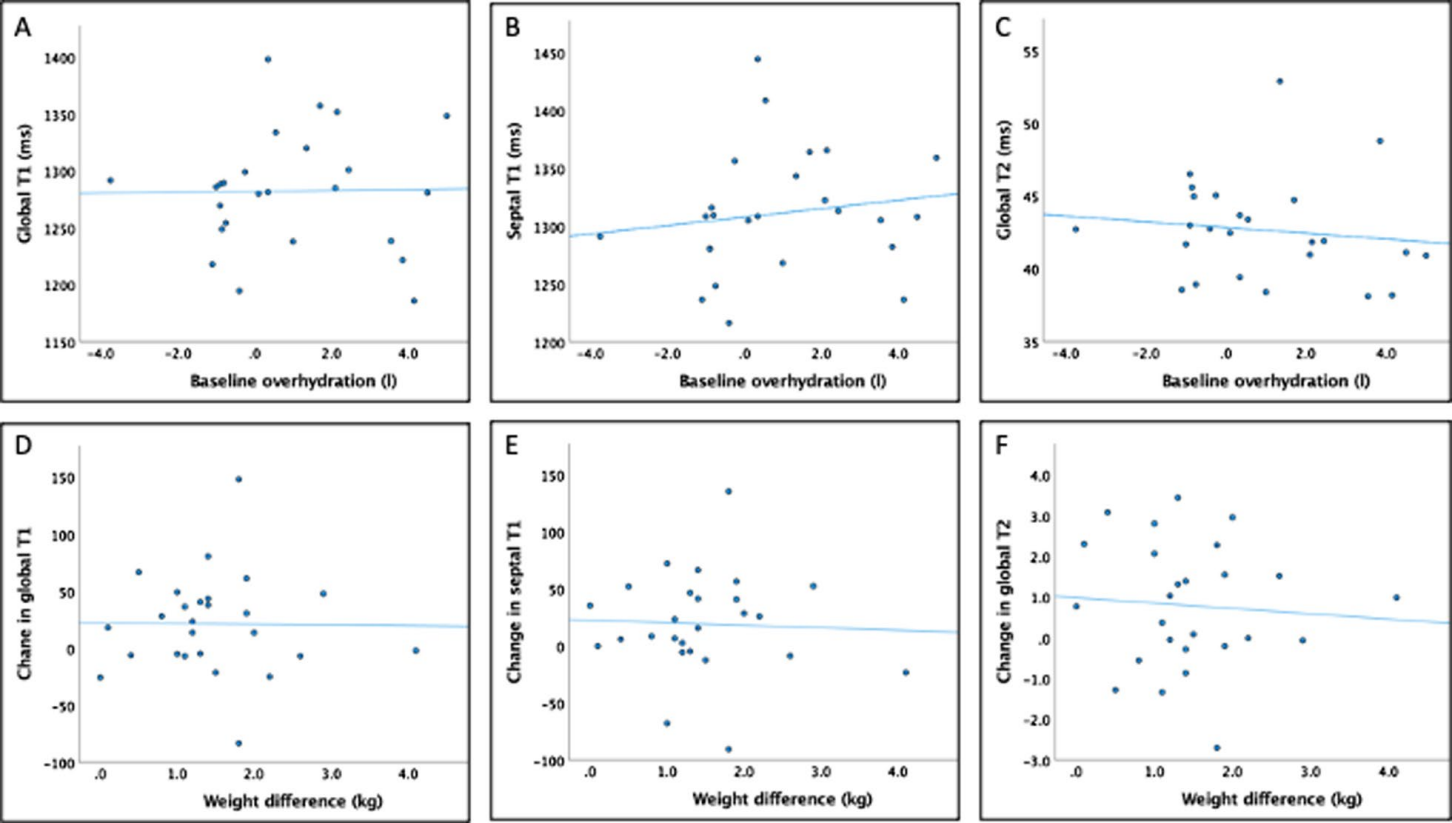
Scatter plots of global T1, septal T1 and global T2 according to baseline over-hydration (**A**–**C** respectively), and the change in global T1, septal T1 and global T2 according to change in weight (**D**–**F** respectively). There was no significant association in any of the comparisons

On linear regression there was no relationship between baseline over-hydration and global native T1, septal T1 or global T2 (Fig. 4). There was also no relationship between the change in global T1, septal T1 or global T2 with ultrafiltration volume (p = 0.88), change in over-hydration (p = 0.87) or change in weight (p = 0.95) (Fig. 4). There was no difference in the mean change in global native T1, septal T1 and global T2 in individuals who did versus did not achieve > 0.5 L reduction in over-hydration (change in global T1: 11.3 (95% CI − 30.2, 52.8), p = 0.58; septal T1: 0.24 (95% CI − 41.8, 42.3), p = 0.99; global T2: − 0.65 (95% CI − 2.1, 0.81), p = 0.37) nor in those with or without > 1.0 kg weight change (change in global T1: 6.3, (95% CI − 39.4, 52.0) p = 0.78; septal T1: 1.2 (95% CI − 45.6, 48.1), p = 0.95; global T2: 0.09, (95%CI − 1.5, 1.7), p = 0.91). Blood pool native T1 correlated with the degree of overhydration measured on bioimpedance at baseline (r^2^ = 0.247, p = 0.013) but there was no association between the change in blood pool T1 and the change in overhydration. There was also no correlation between the change in in myocardial native T1 time and the change in blood pool T1 (r = 0.13, p = 0.54), nor the change in haematocrit (r = − 0.25, p = 0.22). On repeated measures MANCOVA, when the change in blood pool T1 and the change in haematocrit were added as covariates to the comparison of myocardial native T1 time, there was no significant interaction between either covariate and the change in myocardial native T1. Both covariates had small, non-significant contributions to the observed effect (change in blood pool T1 (partial eta squared 0.06, p = 0.72); change in haematocrit (partial eta squared 0.11, p = 0.11), resulting in an adjusted p-value of 0.050 for the comparison of myocardial naïve T1 before and after dialysis. Additional determinants of blood T1 are examined in Additional file 1: Table S4.

### Change in native T1 and T2 parameters by dialysis session

There was no association between the change in global native T1, septal T1 or global T2 and the time from dialysis until repeat CMR (p = 0.80, 0.55 and 0.77 respectively, when mean change in values was compared between morning and afternoon dialysis patients). Eighteen participants were on a morning dialysis schedule, whereas 8 were on an afternoon schedule and thus scanned at different times of day. The mean difference in myocardial native T1 pre/post dialysis was 25 ms in the morning group (n = 8) and 20 ms in the afternoon group (n = 18), with no significant difference between the groups (p = 0.80). When the T1MES phantom was scanned on consecutive days the measured T1 was 1216 ± 8 ms and 1215 ± 13 ms, respectively. The same values for T2 were 80.8 ± 0.9 ms and 80.2 ± 1.9 ms. There was no difference in global T1, septal T1 or global T2 in those participants who experienced symptomatic intradialytic hypotension versus those who did not (p = 0.87, 0.67 and 0.99, respectively). All but 1 participant were prescribed regular intravenous iron therapy. Excluding the 5 participants who received intravenous iron between visit 1 and visit 2 did not change the results (Additional file 1: Table S5).

### Change in LV ejection fraction

**Fig. 5:**
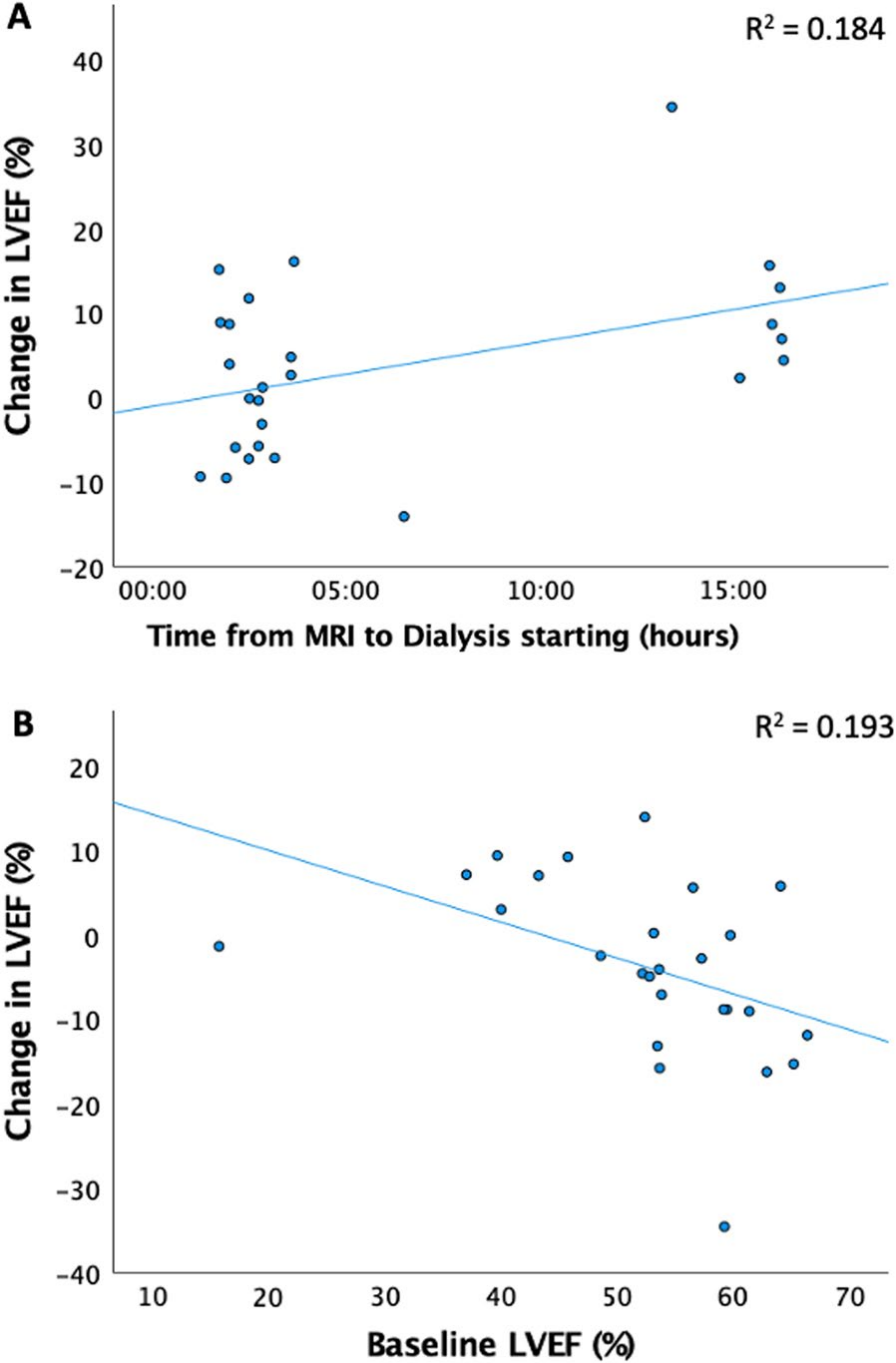
Scatter plots of change in left ventricular ejection fraction (LVEF) [calculated by visit 2 post-dialysis LVEF (%)—visit 1 pre-dialysis LVEF (%)] by baseline LVEF (p = 0.02) (**A**) and time from visit 1 CMR until dialysis (p = 0.04) (**B**)

There was no overall change in LVEF following dialysis (Table 1). Six participants had abnormal LVEF pre-dialysis based on age and sex standardised reference ranges [33]. In 5 of these participants, LVEF improved following dialysis and fluid removal (range 3–9.5%). However, 11 participants with normal LVEF pre-dialysis, had abnormal LVEF post-dialysis. In one participant, a dramatic reduction in LVEF was clearly due to tachy-arrhythmia. In the remaining 10 participants, 4 had minor changes (< 5% difference) that crossed the threshold for age and sex standardised normal values, while 6 had > 5% reduction in LVEF but without obvious association between the change in LVEF and baseline hydration status (visit 1 bioimpedance hydration status ranging from − 3.8 to + 2.5 L). On multivariable linear regression including baseline over-hydration, baseline LVEF, ultrafiltration volume, follow-up over-hydration, over-hydration change, weight change, time from visit 1 until dialysis and time from dialysis until repeat CMR, only baseline LVEF and the time from visit 1 until dialysis significantly associated with the change in LVEF following a backwards elimination approach (baseline LVEF: Beta 0.43, p = 0.02; Time from CMR 1 until dialysis: Beta 0.38, p = 0.04; adjusted r^2^ for the model = 0.30). At lower baseline LVEF, repeat LVEF was more likely to increase, whereas those who had a longer gap between visit 1 CMR and dialysis were more likely to have a reduction in LVEF at visit 2 (Fig. 5).

## Discussion

This prospective study identified significant changes in cardiovascular parameters on 3T CMR in response to haemodialysis with fluid removal. Specifically, LV and RV end-diastolic volumes, stroke volumes, and atrial volumes reduced, as did global native T1, septal native T1 and global T2 times but not LV mass. There was no correlation between the change in these parameters and the change in fluid status measured by bodyweight or bioimpedance. The change in myocardial native T1 time was independent of changes in haematocrit and blood pool T1, suggesting that the observed difference is not explainable by reduced intravascular T1 time. Regardless of whether the change in native T1 time is due to fluid removal, or the dialysis process itself, the present results question the validity of native T1 mapping as a surrogate marker for myocardial fibrosis in patients on haemodialysis.

Native T1 mapping is an appealing potential biomarker for myocardial fibrosis, with proven superiority over volumes, function and late gadolinium enhancement in patients with non-ischaemic dilated cardiomyopathy [34, 35], and encouraging data in patients with CKD [18, 36, 37]. However, there are conflicting results from previous studies exploring the influence of fluid status on native T1 mapping in patients with CKD. Native T1 times do not alter with varying end-diastolic volumes (an indicator of changing fluid status) in patients on dialysis [21]. Similarly, a study comparing 124 dialysis patients to 137 healthy controls found that the increased myocardial native T1 times observed in patients with CKD occurred independently of changes in T2 times, suggesting that fibrosis, rather than fluid, accounts for the differences in T1. However, these patients were scanned the day after dialysis when euvolaemia is most likely [38]. Furthermore, a study of 12 patients found no change in T1 values on 3T CMR immediately post dialysis [39]. These patients had relatively low ultrafiltration volumes (mean 1.1 L) and the lack of effect could be explained by insufficient time to allow for fluid re-equilibration. In the MIDNIGHT study [40], which found significant improvements in native T1 time on 3T CMR with extended hours nocturnal haemodialysis, there was no association between native T1 time and fluid status on bio-impedance body composition monitoring. However, the change in T1 did occur in the presence of increased ultrafiltration volumes in the treatment group, and reduced ultrafiltration volumes in the control group. In contrast, a study of 30 dialysis patients found a significant correlation (r = 0.409) between fluid status and native T1 time on 1.5T CMR [24]. This result could be explained by reverse causality, with patients with more myocardial fibrosis being more prone to fluid overload. Kotecha et al. found global native T1 times on 1.5T CMR reduced from 1085 ms pre-dialysis to 1072 ms post-dialysis in 25 dialysis patients undergoing a 3 h dialysis session with mean 2.0 L ultrafiltration [25]. The present study confirms this result at 3T and supports the conclusion that the abnormal native T1 times observed in patients with CKD can be modulated by dialysis with fluid removal and therefore is not entirely due to fibrosis.

The minimal clinically significant change in native T1 time is difficult to define. Previous studies comparing native T1 in patients on dialysis versus controls found a mean difference of 21 ms on 1.5T [19] and 185 ms on 3T [20]. In the present study, the mean global T1 are greater than the scanner-specific healthy reference range by a mean of 113 ms pre-dialysis and 91 ms post dialysis (Table 1). The mean change in T1 time pre- and post-dialysis was 21 ms. In the MIDNIGHT trial [40], the intervention resulted in a mean reduction in native T1 time of 31 ms (from a mean baseline value of 1270 ms). As another example, in non-CKD patients undergoing aortic valve replacement, native T1 times reduced by an average of 45 ms and were associated with improved prognosis [41]. So while the difference in global T1 observed by this study, and by Kotecha et al. [25], is small, it is within the region of clinically significant difference. Similar magnitude of change has been observed in healthy volunteers and patients with coronary artery disease immediately post exercise, but in this setting native T1 times increased, rather than decreased [42]. In these patients with coronary artery disease, the magnitude of native T1 reactivity correlated with the severity of myocardial perfusion abnormality [42]. This suggests that any change in native T1 times following dialysis is unlikely to be due to dialysis-induced ischaemia (which would cause times to increase).

Native T1 predicts outcome in patients with heart failure [34] and acute myocardial injury [43]. It also has proven diagnostic or prognostic benefit in a range of other conditions including amyloidosis, myocarditis, aortic stenosis, iron overload, and Anderson-Fabry disease [12, 44]. The present results question the on-going consideration of native T1 mapping as a surrogate for myocardial fibrosis in patients on haemodialysis. Studies including myocardial biopsy data will be needed to answer this definitively but would be challenging to justify ethically and difficult to complete. There is an ongoing study correlating native T1 mapping with post-mortem histology in 9 participants (ClinicalTrials.gov Identifier: NCT03586518). For native T1 to proceed as a potential biomarker in CKD patients, it will require longitudinal studies with standardised timing of imaging in relation to dialysis therapy to establish if native T1 has a prognostic role in the CKD population, and if changes in native T1 times correspond with proportional changes in prognosis. If proven, the small changes in native T1 following dialysis may be deemed negligible.

Global T2 times reduced following dialysis with fluid removal, in keeping with previous studies [25]. The native transverse relaxation time (T2) is sensitive to proton (water) binding to macromolecules and proton mobility. Native T2 reflects tissue water content and mobility to a greater extent than native longitudinal relaxation time (T1). Skeletal muscle T2 times also decreased suggesting that the observed myocardial change may be due to reduced total body water content, rather than a myocardial-specific process, but there was no change in hepatic or blood pool T2. The timing of radiofrequency pulse sequence used in T2-weighted images results in increasing signal intensity with increasing water content of tissues [45], and so it is physically plausible that the change in T2 represents reduced tissue oedema. The lack of association between the change in T2 and the change in fluid status is against this, but it still remains the most likely explanation.

There appears to be a complex relationship with regards to parameters of ventricular function and dialysis with fluid removal. A study using intradialytic CMR has previously shown that LVEF drops acutely during dialysis with incomplete recovery evident at 1 h post dialysis [39]. This explains the present observation whereby the timing of dialysis and repeat CMR was a significant factor in predicting repeat LVEF (albeit with a very weak correlation), with those patients on a morning dialysis schedule (and therefore undergoing repeat CMR soon after dialysis) being more likely to have a reduction in repeat LVEF. Paradoxically, in the sub-group of patients with reduced LVEF, previous reports have suggested that dialysis with fluid removal can improve LVEF [25]. In the present study, 5 of the 6 patients with abnormal LVEF at visit 1 had an improvement on repeat LVEF measurement, presumably due to reduced afterload. With regards to clinical practice, CMR scanning should be avoided immediately post-dialysis and serial scanning should be performed at same time in relation to dialysis schedule. Given the differential response in LVEF depending on baseline LVEF, it is conceivable that the wrong dialysis prescription could perpetuate a patient’s cardiac dysfunction and is a reminder of the importance of the individualised medicine in dialysis prescribing. There is increasing interest in the role of LV global longitudinal strain as a potentially superior measure of cardiac function compared to LVEF, especially in patients with CKD [36, 46]. Encouragingly, LV global longitudinal strain did not differ pre- and post-dialysis, suggesting it may give a consistent, volume-independent assessment of cardiac function in dialysis patients.

### Limitations

This study addresses important questions in relation to 3T CMR to inform timing of clinical scanning in relation to dialysis and the potential bias of fluid overload in parametric mapping. The cohort is representative of the wider dialysis population with high prevalence of comorbidity and no changes to their prescribed dialysis session. The number of clinically relevant incidental findings that were identified is striking but is in keeping with previous reports [47]. Fluid assessment was comprehensive and CMR scans were performed utilising state-of-the-art hardware and software. However, there are several limitations. The sample was heterogeneous with regards to timing of scans in relation to dialysis, baseline LVEF (despite attempts to control this by excluding patients with known LV dysfunction) and baseline hydration status, with 10 participants measuring as volume deplete on bioimpedance monitoring at visit 1. We cannot discount a Type 2 error for the lack of correlation between myocardial native T1 and fluid removal. Further, there may have been a differential time-course between changes in fluid status and native T1. The impact of 1 L fluid removal is likely to have differential effect on the myocardium if the starting state is volume overload, as opposed to volume depletion. Nevertheless, there was no apparent difference in change in native T1 times according to baseline hydration status. The study could have been improved by inclusion of a control group who underwent dialysis without fluid removal. Further work is warranted.

## Conclusion

Acute changes in cardiac volumes and myocardial composition are detectable on 3T CMR following haemodialysis with fluid removal. Accordingly, the timing of clinical CMR scanning in relation to a patient’s dialysis schedule is crucial, particularly if serial scanning is required. Small, but significant, reductions in global myocardial T1 and T2 relaxation times were observed after dialysis suggesting that the abnormal native T1 signal in patients undergoing haemodialysis is not entirely due to fibrosis. The exact mechanism for the reduction in native T1 is unclear. Despite the lack of association with the change in native T1 and the change in fluid status, alterations in tissue oedema remain the most likely explanation, albeit removal of uraemic factors or the haemodynamic effects of dialysis itself may also contribute. Future studies examining the prognostic capabilities of native T1 in CKD populations are still warranted but will require careful standardisation of imaging schedules and awareness of the potential confounding effect of fluid status and the dialysis process.

## Supplementary Information

**Additional file 1.** **Supplementary material: S1.** Baseline characteristics of participants. **S2.** Summary of clinically significant incidental findings. **S3.** Intra- and inter-observer reproducibility for cardiovascular MRI parameters. **S4.** Determinants of blood pool native T1. **S5.** Analyses exploring the potential influence of intravenous iron therapy on native T1.

## Data Availability

Available via the corresponding author upon reasonable request.

## Abbreviations

CKD: Chronic kidney disease
CMR: Cardiovascular magnetic resonance
CVD: Cardiovascular disease
ECG: Electrocardiogram
LA: Left atrium/left atrial
LV: Left ventricle/left ventricular
LVEF: Left ventricular ejection fraction
RA: Right atrium/right atrial
RV: Right ventricle/right ventricular
