# Supplementary material for: Myocardial changes on 3T cardiovascular magnetic resonance imaging in response to haemodialysis with fluid removal
Source: J Cardiovasc Magn Reson. 2021 Nov 11;23:125. doi: 10.1186/s12968-021-00822-4 (PMC8580743; doi:10.1186/s12968-021-00822-4)
Supplement: Supplementary file 1 — Additional file 1. Supplementary material: S1. Baseline characteristics of participants. S2. Summary of clinically significant incidental findings. S3. Intra- and inter-observer reproducibility for cardiovascular MRI parameters. S4. Determinants of blood pool native T1. S5. Analyses exploring the potential influence of intravenous iron therapy on native T1. [file 12968_2021_822_MOESM1_ESM.docx]

**Additional material**

**Additional file Table S1.** Baseline characteristics. Values are displayed as count (percentage), mean +/- standard deviation or median (interquartile range), as appropriate.

| Age, years | 64.7 ± 9.4 |
| --- | --- |
| Male | 16 (62%) |
| Primary renal diagnosis   - Diabetes - Glomerulonephritis - Polycystic - Tubulo-interstitial nephritis - Unknown - Other | 8 (31%)  6 (23%)  1 (4%)  2 (8%)  4 (15%)  5 (19%) |
| - Duration of renal replacement therapy, years | 2.01 (1.34, 4.04) |
| Dialysis schedule   - Morning - Afternoon | 18 (69%)  8 (31%) |
| Dialysis access   - Catheter - Arterial venous fistula - Arterio-venous graft | 9 (5%)  12 (46%)  5 (19%) |
| Body mass index (kg/m2) | 31.7 (27.2, 36.6) |
| Smoking   - Previous - Current | 8 (31%)  1 (4%) |
| Hypertension | 24 (92%) |
| Previous myocardial infarction | 3 (12%) |
| Angina | 5 (19%) |
| Stroke | 7 (27%) |

**Additional file Table S2:** summary of clinically significant incidental findings. These findings were detected on the clinical radiology report which was issued by a consultant radiologist for each research scan. Participants were informed and appropriate follow-up arranged in each case.

| N=26 |  |
| --- | --- |
| 1 | Renal cancer requiring nephrectomy |
| 1 | Metastatic bladder cancer |
| 1 | Aspiration pneumonia |
| 1 | Decompensated severe left ventricular systolic dysfunction |
| 1 | Pulmonary nodule requiring follow-up |
| 1 | Pleural plaques |

Additional **file** table S3: Intra- and inter-observer reproducibility for cardiovascular MRI (CMR) parameters assessed by intraclass correlation coefficient (ICC) (two-way mixed effect, absolute agreement). Performed on a random sample of 11 participants representing >20% of total cohort.

| **CMR parameter** | **Intra-observer ICC** | **Inter-observer ICC** |
| --- | --- | --- |
| LV myocardial mass (g) | 0.986 | 0.947 |
| LV end diastolic volume (ml) | 0.998 | 0.960 |
| LV end systolic volume (ml) | 0.975 | 0.934 |
| LV stroke volume (ml) | 0.968 | - |
| LV ejection fraction (%) | 0.870 | 0.863 |
| LV global longitudinal strain (%) | 0.969 | 0.903 |
| LV global circumferential strain (%) | 0.968 | - |
| LV global radial strain (%) | 0.956 | - |
| LV thickness (mm) | 0.887 | - |
| RV end diastolic volume (ml) | 0.983 | - |
| RV end systolic volume (ml) | 0.983 | - |
| RV stroke volume (ml) | 0.927 | - |
| RV ejection fraction (%) | 0.897 | - |
| RV global longitudinal strain (%) | 0.632 | - |
| RV global radial strain (%) | 0.608 | - |
| minimum LA volume (ml) | 0.927 | - |
| maximum LA volume (ml) | 0.995 | - |
| minimum RA volume (ml) | 0.981 | - |
| maximum RA volume (ml) | 0.987 | - |
| Global native T1 (ms) | 0.989 | 0.949 |
| Septal native T1 (ms) | 0.934 | 0.937 |
| Blood pool native T1 (ms) | 0.958 | - |
| Skeletal muscle native T1 (ms) | 0.694 | - |
| Liver native T1 (ms) | 0.989 | - |
| Global T2 (ms) | 0.962 | 0.953 |
| Blood pool T2 (ms) | 0.831 | - |
| Skeletal muscle T2 (ms) | 0.921 | - |
| Liver T2 (ms) | 0.978 | - |

*Abbreviations:*

*LV = left ventricular*

*LA = left atrial*

*RA = right atrial*

*RV = right ventricular*

Additional **file Table** S4: Determinants of blood pool T1

There was no significant difference in blood pool native T1 pre and post dialysis (Pre 1956.7 ms (+/- 67.8); Post 1934.7 (+/- 72.9); p=0.08). Blood pool native T1 correlated with the degree of overhydration measured on bioimpedance at baseline (r2 = 0.247, p=0.013) but there was no association between the change in blood pool T1 and the change in overhydration. There was no correlation between the change in blood pool T1 and the change in myocardial native T1 (r=0.13, p=0.54). Previous studies have suggested a high degree of correlation between blood pool native T1 and biochemical parameters (*S Rosmini, H Bulluck, A Abdel-Gadir et al, The Effect of Blood Composition on T1 Mapping, J Am Coll Cardiol Img. 2019. 12 (9);1888-1890)*. In the present study, there was no correlation between blood pool native t1 and the following parameters: haematocrit (p=0.84), haemoglobin (p=0.63), creatinine (p=0.69), iron (p=0.62), transferrin saturation (p=0.72), serum albumin (p=0.77), triglycerides (p=0.72), cholesterol (p=0.63), LDL (p=0.76) and HDL (p=0.73). The following table shows the mean change in blood parameters pre and post dialysis:

|  | Pre-dialysis | Post dialysis |
| --- | --- | --- |
| Haemoglobin (g/L) | 115 (15) | 115 (14) |
| Haematocrit (L/L) | 0.363 (0.046) | 0.363 (0.045) |
| Sodium (mmol/L) | 137 (4) | 137 (2) |
| Potassium (mmol/L) | 5.4 (0.9) | 4.6 (0.7) |
| Urea (mmol/L) | 21.1 (4.7) | 10.5 (3.3) |
| Creatinine (umol/L) | 815 (244) | 507 (205) |
| Albumin (g/L) | 35 (4) | 34 (4) |
| Ferritin (ng/mL) | 465 (346) | 502 (358) |
| Iron (umol/L) | 11 (6) | 15 (10) |
| Transferrin (g/L) | 1.9 (0.4) | 1.9 (0.4) |
| Transferrin Saturation (%) | 24 (12) | 32 (22) |
| Triglycerides (mmol/L) | 2.7 (2.3) | 2.2 (2.1) |
| Cholesterol (mmol/L) | 4.6 (1.5) | 4.2 (0.9) |
| High density lipoprotein (mmol/L) | 1.2 (0.5) | 1.3 (0.4) |
| Low density lipoprotein (mmol/L) | 2.1 (0.8) | 2.0 (0.8) |

Values displayed as mean (standard deviation).

Additional material S5: Analyses exploring the potential influence of intravenous iron therapy. Excluding the 5 participants who received intravenous iron between visit 1 and visit 2 did not change results: a significant reduction in global T1 was still observed (mean 22.3ms, p=0.04).

The native T1 times for the 5 patients who received intravenous iron between scans is included in the table below:

| Participant | Global native 1 pre-dialysis | Global native T1 post dialysis | Dose of intravenous iron sucrose received |
| --- | --- | --- | --- |
| 1 | 1218.1 | 1200.1 | 150 mg |
| 2 | 1289.6 | 1240.3 | 25 mg |
| 3 | 1281.0 | 1233.1 | 100 mg |
| 4 | 1221.7 | 1226.5 | 100 mg |
| 5 | 1299.14 | 1324.1 | 50 mg |

**List of Abbreviations**

- CKD = chronic kidney disease
- CMR = cardiovascular magnetic resonance imaging
- CVD = cardiovascular disease
- ICC = intra-class correlation
- IQR = interquartile range
- *LA = left atrial*
- LV = left ventricular
- LVEF = left ventricular LV ejection fraction
- *RA = right atrial*
- ROI = regions of interest
- RV = right ventricular
- SD = standard deviation
- TE = echo time
